# Evidence for selection and spatially distinct patterns found in a putative zona pellucida gene in Pacific cod, and implications for management

**DOI:** 10.1002/ece3.8284

**Published:** 2021-11-30

**Authors:** Ingrid Spies, Daniel P. Drinan, Eleni L. Petrou, Rory Spurr, Carolyn Tarpey, Theodore Hartinger, Wes Larson, Lorenz Hauser

**Affiliations:** ^1^ Resource Ecology and Fisheries Management Division Alaska Fisheries Science Center Seattle Washington USA; ^2^ School of Aquatic and Fishery Sciences University of Washington Seattle Washington USA; ^3^ Ted Stevens Marine Research Institute Alaska Fisheries Science Center/Auke Bay Laboratory Juneau Alaska USA

**Keywords:** fisheries management, Pacific cod, selection, zona pellucida

## Abstract

Genetic differentiation has been observed in marine species even when no obvious barriers to gene flow exist, and understanding such differentiation is essential for effective fisheries management. Highly differentiated outlier loci can provide information on how genetic variation might not only contribute to local adaptation but may also be affected by historical demographic events. A locus which aligned to a predicted zona pellucida sperm‐binding protein 3 gene (ZP3) in Atlantic cod (*Gadus morhua*) was previously identified as the highest outlier based on *F*_ST_ in a RADseq study of Pacific cod (*Gadus macrocephalus*) across the West Coast of North America. However, because of the limited length of the RAD sequence and restricted geographic area of sampling, no conclusion on the functional significance of the observed variation was possible. In other marine species, ZP3 is involved in reproductive isolation, local adaptation, and has neofunctionalized as an antifreeze gene, and so it may provide important insights in functional population structure of Pacific cod. Here, we sequenced a 544‐bp region of ZP3 in 230 Pacific cod collected from throughout their geographic range. We observed striking patterns of spatial structuring of ZP3 haplotypes, with a sharp break near Kodiak, Alaska, USA where populations within ~200 km of each other are nearly fixed for different haplotypes, contrasting a pattern of isolation by distance at other genetic markers in this region (*F*_ST_ = 0.003). Phylogenetic analysis of ZP3 haplotypes revealed that the more southern haplotypes appear to be ancestral, with the northern haplotype evolving more recently, potentially in response to a novel selective pressure as Pacific cod recolonized northern latitudes after glaciation. The sharp break in haplotype frequencies suggests strong selective pressures are operating on small spatial scales and illustrates that selection can create high divergence even in marine species with ample opportunities for gene flow.

## Graphical abstract

We document a sharp break in haplotype frequencies for a putative sperm‐binding protein zona pellucida subunit 3 in Pacific cod. Results suggest strong selective pressures are operating on small spatial scales and illustrate that selection can create high divergence even in marine species with ample opportunities for gene flow.

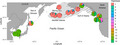

## 1 INTRODUCTION

A primary objective of fisheries management in the marine realm involves understanding intraspecific genetic diversity. While the ocean offers few apparent boundaries to long distance dispersal at any life stage, population divergence has been observed in many species (Hauser & Carvalho, 2008). Accounting for differences among local populations and understanding the mechanisms behind divergence are key to managing for optimal yield (Spies et al., 2015). Genetic differentiation across the genome is the result of complex interactions between genetic drift, selection, mutation, migration, and recombination (Nosil et al., 2009). Natural selection may override the homogenizing effects of gene flow, not only at loci under selection but also at linked loci, often leading to ‘genomic islands of divergence’, that is, sections of the genome where differentiation is much higher than elsewhere (Nosil et al., 2009; Via, 2012). Outlier loci with particularly high levels of divergence can provide the opportunity to evaluate population structure, examine selective forces that maintain population diversity (Petrou et al., 2021), identify migrating individuals (Fisher et al., 2021), and estimate population contributions to mixed fisheries (Bekkevold et al., 2015; Dahle et al., 2018).

Pacific cod (*Gadus macrocephalus*) provide an interesting case study to investigate the effects of selective differentiation. Pacific cod are found demersally throughout the Western Pacific as far south as the Korean Peninsula, northward to the Bering and Chukchi Sea, throughout the Aleutian Islands and southward along the eastern Pacific as far as northern California. Populations at the southern edges of both the eastern and western Pacific show deep genetic divergence from northern populations (Canino et al., 2010), and studies of neutrally evolving loci have identified moderately genetically differentiated populations of Pacific cod (*F*_ST_ < 0.02) throughout the North Pacific that display a general isolation‐by‐distance pattern indicating somewhat limited dispersal (e.g., Cunningham et al., 2009; Drinan et al., 2018; Smirnova et al., 2018, 2019).

Pacific cod undertake annual feeding migrations that are not well characterized but they return to their natal spawning areas during winter months to reproduce (Neidetcher et al., 2014; Rand et al., 2014). While many of the genetic differences between Pacific cod populations can be explained by reproductive isolation caused by strong homing behavior, the mechanisms underlying selection have not been specifically explored. Such selective processes are increasingly relevant to management, given recent ocean warming events that have resulted in steep declines in Gulf of Alaska populations (Barbeaux, Holsman, et al., 2020), and anomalous summer feeding migrations into the northern Bering Sea (Spies et al., 2020). These events emphasize sensitivity to temperature related to growth and survival in Pacific cod (Barbeaux, Holsman, et al., 2020; Hurst et al., 2009; Laurel et al., 2008). Furthermore, the broad species range of Pacific cod, from temperate climates of Korea (34°N) and the Pacific Northwest (47°N) to the arctic and subarctic conditions of the Bering and Chukchi Seas (>58°N) suggests that local populations of cod are adapted to thermal profiles specific to their habitat.

One of the prime candidates for a gene under selection detected in a previous restriction site‐associated DNA sequencing (RADseq) study (Table A1, Drinan et al., 2018) was a gene encoding for the zona pellucida sperm‐binding protein 3 (ZP3) predicted in Atlantic cod (*Gadus morhua*). ZP3 egg coat proteins are part of a multigene family that is central to reproductive isolation and prevention of polyspermy in mammals (Shu et al., 2015), but that may have more complicated functions in fish (Conner et al., 2005; Wassarman, 2008). Zona pellucida egg coat proteins function in a range of capacities such as protecting the embryo and mediating fertilization. Furthermore, duplicated copies of zona pellucida genes have neofunctionalized to produce antifreeze proteins in the eggs of Antarctic notothenioid fishes (Cao et al., 2016; Gupta et al., 2012). In general, genes coding for proteins on gamete surfaces appear to evolve quickly and may be subject to positive selection (Palumbi, 2009).

In this study, we used DNA sequencing to assess geographic variation and examine signals of selection in the putative ZP3‐coding gene among samples representative of the range of Pacific cod. We also integrated ZP3 data with a previously published RADseq dataset (Drinan et al., 2018) to compare neutral variation with variation at the putative ZP3‐coding region. Our study had three specific aims: (i) to determine spatial patterns of differentiation at ZP3 across most of the range of Pacific cod, (ii) to confirm elevated differentiation among Pacific cod populations at ZP3 compared to neutral markers, and (iii) to test for signals of selection at this gene. We hypothesized that patterns of diversification in this putative ZP3 gene would provide further support for strong divergent selection across small spatial scales and would provide information about the functional significance of this gene.

## 2 MATERIALS AND METHODS

### 2.1 DNA extraction, DNA sequencing, screening for variation

Fin clips from Pacific cod were collected from 16 spawning locations across the species range and preserved in 95% ethanol (Table 1). Samples were taken during the winter spawning season (December through May) to take advantage of spawning site fidelity and avoid sampling of population mixtures. Samples were taken from a range of years from 2003 to 2019. Pacific cod populations have demonstrated genetic stability over time; therefore, samples collected over a range of years were expected to be representative of the population‐specific allele frequencies (Cunningham et al., 2009). DNA was extracted using Qiagen Blood and Tissue kits following manufacturers’ instructions (Qiagen, Inc.).

**TABLE 1:** Pacific cod (*Gadus macrocephalus*) samples used in this study, listed in clockwise order around the Pacific Rim, including location name, region, date sampled, coordinates, and number of individuals sequenced (*n*)

Location	Region	Date	Latitude	Longitude	*n*
Geoje, Korea	Western Pacific	Jan. 2015	34°51'N	128°35'E	10
Yellow Sea, Korea	Western Pacific	Feb. 2015	35°12'N	124°40'E	2
Jukbeon, Korea	Western Pacific	Dec. 2007	37°04'N	129°25'E	9
Japan (Sea of Okhotsk)	Western Pacific	May 2005	44°20'N	145°52'E	12
Near Islands	Bering	Feb. 2005	52°34'N	174°17'E	22
Kiska Island	Bering	Mar. 2005	51°48'N	177°47'E	22
Pervenets Canyon	Bering	Mar. 2016	59°21'N	177°13'W	21
Adak Island	Bering	Mar. 2006	51°40'N	176°36'W	7
Unimak Pass	Bering	Feb. 2018	54°35'N	165°15'W	14
Shumagin Is.	Bering	Mar. 2019	55°15'N	159°30'W	23
Kodiak Island	Bering	Mar. 2003	57°48'N	152°31'W	26
Prince William S.	Eastern Pacific	Mar. 2012	60°32'N	147°04'W	12
Hecate Strait	Eastern Pacific	Mar. 2004	53°13′N	130°57′W	13
Strait of Georgia	Eastern Pacific	Apr. 2003	48°54'N	123°06'W	6
Puget Sound	Eastern Pacific	Mar. 2003	47°35'N	122°30'W	8
Puget Sound	Eastern Pacific	May 2012	48°14'N	122°40'W	1
Washington Coast	Eastern Pacific	Feb. 2005	47°55'N	125°33'W	22

Note

Sample names refer to a nearby landmark. Sampling locations are considered within three broad regions: Western Pacific (including Korea and Japan), Bering Sea (including Aleutian Islands, Bering Sea, and Western Gulf of Alaska), and Eastern Pacific (eastern Gulf of Alaska southward to Washington State). Samples from Puget Sound collected in 2003 and 2012 were combined for all analyses due small sample sizes.

Four sets of DNA primers were designed to screen for variation in four exon regions aligning with the ZP3‐coding region in Pacific cod (Table A2). All primers were designed using Primer3 v. 0.4.0 (Koressaar & Remm, 2007). Five individuals of Pacific cod from the Hecate Strait, Prince William Sound, Washington (WA) Coast, and Kodiak Island collections were sequenced for initial screening (Table 1). Polymerase chain reactions (PCRs) for the first four sets of primers were carried out in 40 μl volume with 10 μM forward and reverse primers using the Qiagen Taq PCR Master Mix kit and approximately 200 ng DNA (Table A2). Thermal cycling conditions were 95°C for 15 min, followed by 35 cycles of 94°C for 30 s, *T*_M_ (Table A2) for 90 s, and 72°C for 60 s, and a final elongation at 72°C for 30 s.

Following this initial screening, a single set of primers was designed to focus on variable regions observed in Exons 2, 3, and 4 in the ZP3‐coding region (primer ZP_GM, Table A2). PCRs for the variable region (primer ZP_GM, Table A2) were carried out in a 25‐µl volume, with Phusion 5× buffer (New England Biolabs), 10 mM dNTPs, 10 µM forward and reverse primers, 0.2 µl Phusion *Taq* polymerase, and approximately 200 ng DNA. Thermal cycling conditions were 98°C for 30 s, followed by 5 cycles of 98°C for 10 s, 63°C–59°C touchdown for 30 s (−1°C each cycle for 5 cycles) and 72°C for 30 s, and then by 30 cycles of 98°C for 10 s, 58°C for 30 s, and 72°C for 30 s, and concluded at 72°C for 5 min.

A total of 230 Pacific cod were sequenced to screen 544 bp of the variable region of ZP3 using primers ZP_GM (Table 1). Sanger sequencing was performed bidirectionally using forward and reverse primers at MCLAB (320 Harbor Way). Contigs were aligned using Sequencher v. 5.0 (Gene Codes Corporation) and scores below 80% quality were discarded. Sequence calls were double checked and confirmed by two readers when ambiguities were present. Consensus sequences were aligned in BioEdit v. 7.2 (Hall, 1999). Sanger DNA sequence data were transformed from unphased sequences to fasta files with two haplotypes for all individuals. Haplotypes for ambiguous (heterozygous) nucleotides at ZP3‐segregating sites were inferred using Bayesian methodology with a priori expectations based on coalescent theory in PHASE v2.1.1 and SeqPHASE (Flot, 2010; Stephens et al., 2001). The most likely pair of haplotypes was selected for each individual based on the highest posterior probability.

Sequences were aligned to two Atlantic cod genomes in GenBank using BLASTn to ensure our sequenced region aligned with ZP3 and to identify synonymous and nonsynonymous substitutions (Sayers et al., 2019). The Atlantic cod genome is the most closely available genome to Pacific cod (Árnason & Halldórsdóttir, 2019). The gadMor3.0 (RefSeq GCF 902167405.1) assembly of Atlantic cod originates from an individual from the North East Arctic Sea, and thus likely from a cold‐adapted type, while a genome assembly available from a more southerly Celtic Sea cod likely represents a warm‐adapted genotype (Kirubakaran et al., 2020). We chose to test alignments in the northern and southern Atlantic cod reference genomes to examine whether ZP3 would differ among warm‐ and cold‐adapted individuals. The predicted zona pellucida sperm‐binding protein 3 (ZP3) spans a 3407‐bp DNA sequence of the gadMor3.0 annotated genome. It is located on linkage group 9 (LG09) in that assembly and spans nucleotides 2,454,601–2,458,007 (GenBank Accession ID: GCF_902167405.1, NC_044056.1). This region was also identified in the gadMor_Celtic assembly (Kirubakaran et al., 2020) on linkage group 9 from 23,598,961 to 23,602,367. Our 544‐bp DNA sequence data were aligned using BioEdit to the reverse complemented zona pellucida sperm‐binding protein 3‐like of Atlantic cod based on the gadMor3.0 (Gene ID: 115551355, updated Nov. 22, 2020) assembly on chromosome 9 to identify intron and exon regions. The ZP3 regions of gadMor_Celtic and gadMor3.0 were identical at all segregating sites in Pacific cod so further comparisons to the Atlantic cod genome were limited to gadMor3.0.

### 2.2 Spatial structuring, basic statistics

Pacific cod is believed to have originated from an Atlantic cod ancestor that moved into the Pacific Ocean (Árnason & Halldórsdóttir, 2019); therefore, Pacific cod haplotypes more similar to gadMor3.0 were assumed to be ancestral. Haplotype networks were generated in the R package *pegas* to visualize relationships among DNA sequences and make inferences about biogeography and population history (Paradis, 2010), using R version 4.03 (R Core Team, 2019). Pairwise distances were computed among the 14 unique DNA haplotypes and the complementary 544‐bp sequence in Atlantic cod, based on gadMor3.0, to understand which haplotypes appeared most similar to Atlantic cod, using the R package *ape* v.5.4.1 (Paradis & Schliep, 2019) and *phangorn* (Schliep et al., 2017). We selected best model using the Bayesian Information Criterion (BIC) because BIC places a higher penalty on model complexity than Akaike Information Criterion (AIC Akaike, 1978).

*G*_ST_ was selected to measure the variance in allele frequencies among populations because it is a standard measure of genetic differentiation for DNA sequence data (Nei, 1973). *G*_ST_ can be dependent on sample size, so we tested relationship between sample size and *G*_ST_ using a simple regression. Global and pairwise *G*_ST_ were calculated using the R package *mmod*, and confidence intervals were generated with 1000 bootstrap replicates (Winter, 2012). Dendrograms of hierarchical clusters of sample collections were generated based on pairwise *G*_ST_ using the *pvclust* package, and support for each cluster was based on 10,000 bootstrap replicates (Suzuki & Shimodaira, 2006). Dendrogram probabilities were presented as bootstrap probabilities (BP, green) and approximately unbiased values (AU, red), which may be more accurate than bootstrap values (Suzuki & Shimodaira, 2006). Haplotype (*h*) and nucleotide diversity (*π*) were calculated using the R package *pegas*. We calculated expected heterozygosity (*H*_e_) per population using the estimator *θ*_H_, which corrects for potential overestimation when few loci are present, in Arlequin v. 3.5 (Excoffier & Lischer, 2010). Observed heterozygosity (*H*_o_) was calculated as the proportion of heterozygous individuals (individuals with different ZP3 haplotypes), out of the total number sequenced. *F*_IS_ was calculated by haplotype as *F*_IS_ = 1–*H*_o_/*H*_e_ (Nei, 1986). Fisher’s exact test was used to test for significant deviations from Hardy–Weinberg equilibrium for each collection using the R package *stats* (fisher.exact) and 10^7^ Markov Chain replicates. We enumerated the number of transitions and transversions over all haplotypes.

### 2.3 Statistical tests of selective neutrality

Nonsynonymous (d*_N_*) and synonymous (d*_S_*) substitutions were enumerated based on open reading frames from the predicted *Gadus morhua* zona pellucida sperm protein 3‐like sequence (GenBank Accession number XM_030365288). Several tests were performed to test for selective neutrality. The Ewens–Watterson homozygosity test for selective neutrality and an exact test for selective neutrality based on Ewens’ sampling theory were performed in Arlequin v. 3.5 with 16,000 simulated samples. The exact test based on Ewens’ sampling theory (Slatkin, 1996) compares the probability of the observed sample with that of a random neutral sample with the same number of alleles and identical size, while the Ewens–Watterson homozygosity test (Watterson, 1986) is based on Ewens sampling theory (Ewens, 1972). For these statistics, the observed haplotype frequencies (*F*) were calculated from the data and the expected *F* was generated from a simulated random neutral sample with the same number of haplotypes. For the exact test and the Ewens–Watterson homozygosity test, small probability values, *p* = Pr (*F*_EXP_ ≤ *F*_OBS_) indicate balancing selection (*p* ≤ .05 considered significant and *p* ≤ .10 marginally significant), while large values indicate directional or divergent selection (*p* ≥ .95 considered significant and *p* ≥ .9 marginally significant); note that these *p* values are not statistical test probabilities but the placement of an observed value in a distribution of values expected under neutrality. Tajima’s *D* test was calculated as an additional measure of statistical neutrality in *pegas,* and *p*‐values were calculated based on the assumption that *D* follows a beta distribution scaled from 0 to 1. A negative value of Tajima’s *D* indicates population size expansion, such as after a bottleneck or a selective sweep and/or purifying selection, while a positive value indicates balancing selection or decrease in population size. Tajima’s *D* was not calculated over multiple collections, as an admixed sample can result in the appearance of an excess of low‐frequency mutations (Stajich and Hahn, 2005). Because changes in population size can confound interpretation of Tajima’s *D*, we also calculated the Ramos‐Onsins–Rozas test (*R*_2_) for detecting changes in population size (Ramos‐Onsins & Rozas, 2002, Sano & Tachida, 2005). In this test, a small *p*‐value indicates population increase and large *p*‐value indicates population decline.

### 2.4 Analyses with previously published RADseq dataset

We re‐analyzed a previously published RADseq dataset that included 6,425 SNPs, hereafter referred to as the RADseq dataset (Drinan et al., 2018), downloaded from https://datadryad.org/stash/dataset/doi:10.5061/dryad.402sb71 to compare ZP3 sequence data with a larger set of mostly neutral SNPs mapped to the Atlantic cod genome (gadMor3.0). First, the RADseq dataset was aligned with gadMor3.0 using bowtie2 v. 2.2.6 software (–sensitive alignment) (Langmead & Salzberg, 2012). Loci were retained for this analysis if they had mapping quality ≥20. Next, we used the RADseq dataset to calculate linkage disequilibrium across linkage group 9 to determine whether it was significantly increased in the region associated with the ZP3 SNPs, because linkage disequilibrium is significantly increased in regions containing genomic inversions. Linkage disequilibrium along LG09 was estimated using the R package *genetics* v. 1.3.8 (Warnes et al., 2019), and per‐locus *F*_ST_ was measured in the R package *genepop* (Rousset, 2008).

We also calculated pairwise *F*_ST_ (Nei, 1987) between Kodiak Island and Prince William Sound collections using the *R* packages *adegenet* and *hierfstat* (Goudet, 2005; Jombart et al., 2008), to compare *F*_ST_ at the ZP3 region with other genomic locations. The short read sequence from Drinan et al. (2018) contained two SNPs at positions 313 and 339 (Table 2, Table 3). Only the SNP at site 339 was selected (randomly) in the final RADseq dataset from Drinan et al. (2018). We added the eight additional SNPs from the current dataset to compare *F*_ST_ across the genome. Dendrograms of hierarchical clusters of sample collections were generated based on pairwise *F*_ST_, similar to the methodology used for pairwise *G*_ST_, for four of the same sample collections used in both studies (Adak, PWS, Hecate, Washington Coast, Table 1, Drinan et al., 2018) and Puget Sound and Unimak samples taken in different years.

**TABLE 2:** The position of each segregating site in the 544‐bp sequence of Pacific cod (*Gadus macrocephalus*), and its position on the zona pellucida sperm‐binding protein 3 DNA‐coding region of linkage group 9 in the gadMor3.0 alignment (NC_044056.1). Also shown are its codon position (1, 2, or 3), location in an intron or exon, and resulting amino acid change

Position in sequence	Position on gadMor3.0	Reading frame	Intron or Exon	Amino acid change
17	2,455,229	–	Intron	
313	2,455,525	2	Exon 3	AGG (Arg) or ATG (Met)
339	2,455,551	1	Exon 3	GAC (Asp) or AAC (Asn)
447	2,455,649	–	Intron	
448	2,455,650	–	Intron	
449	2,455,651	–	Intron	
451	2,455,653	–	Intron	
452	2,455,654	–	Intron	
542	2,455,754	2	Exon 4	TAC (Tyr) or TTC (Phe)

**TABLE 3:** There were 14 inferred haplotypes of Pacific cod (*Gadus macrocephalus*), at nine segregating sites at 17, 313, 339, 447, 448, 449, 451, 452, 542 bp along the 544‐bp sequence

Designation	Haplotype	*N*	*d*	d*_N_*	d*_S_*
1	GTATATGCT	265	0.0436	3	1
2	GTATATGCA	5	0.0416	2	1
3	GGATATGCT	3	0.0416	2	1
4	GGGTATGCT	10	0.0397	1	1
5	GGGTATGCA	3	0.0377	0	1
6	GGGCGATTT	1	0.0494	1	6
7	GGGCGATTA	1	0.0475	0	6
8	ATGTATGCT	11	0.0397	2	0
9	ATGTATGCA	1	0.0378	1	0
10	AGATATGCT	13	0.0397	2	0
11	AGATATGCA	5	0.0378	1	0
12	AGGTATGCT	6	0.0377	1	0
13	AGGTATGCA	99	0.0358	0	0
14	AGGCGATTA	37	0.0455	0	5
gadMor3.0	AGGTATGCA	–	–	–	–
gadMor_Celtic	AGGTATGCA	–	–	–	–

Note

The gadMor3.0 and gadMor_Celtic haplotypes are shown below the Pacific cod haplotypes. *N* refers to the number of each haplotype observed, for 230 diploid individuals (460 sequences), the pairwise distance (F81, Felsenstein, 1981) calculated between each haplotype and the ZP3 sequence from Atlantic cod, and the number of nonsynonymous (d*_N_*) and synonymous (d*_S_*) substitutions in each haplotype compared to gadMor3.0. The haplotype designations listed here are used throughout. Dashes indicate not applicable.

## 3 RESULTS

### 3.1 DNA sequencing, screening for variation

Our initial screening efforts revealed no variation in DNA sequence within Exons 1, 5, 6, or 8, which were sequenced using primers ZP_L1, ZP_L3, or ZP_L4. Variation was only observed in Exons 2, 3, and 4 in sequences generated using primers ZP_L2 (GenBank Accession numbers MW468336–MW468402). New primers (ZP_GM) were designed to sequence variation observed in Exons 2, 3, and 4 because primer ZP_L2 did not amplify in all collections (Table 1, Table A2). Data for the 230 Pacific cod sequenced using primers ZP_GM are available at Genbank (Accession numbers MW468106–MW468335). The number of individuals sequenced per collection ranged from 1 to 26 (Table 1). Samples from Puget Sound collected in 2003 and 2012 were combined for all analyses due to small sample sizes.

Sequences were trimmed to a final length of 544 bp, which included 40 amino acid residues from Exon 2, 35 amino acid residues from Exon 3, and 4 amino acid residues from Exon 4. There were nine variable sites; three were located in exons and six in introns (Table 2). Variable sites at location 313 and 339 bp were located in Exon 3 and the variable site at location 542 was in Exon 4 (Table 2).

All Bayesian probabilities for inference of heterozygote phasing were ≥ 0.9, with the exception of one individual. There were three possible haplotypes for a sample from Prince William Sound (Genbank Accession MW468263), with probabilities of .272, .256, and .469. This was a unique case with two heterozygous sites and probabilities were constrained to sum to 1. While *p* = .469 appeared low, it was twice as likely as the other two haplotypes and was, therefore, selected. Phased sequences are available at https://doi.org/10.5061/dryad.wdbrv15q7.

### 3.2 Spatial structuring

**FIGURE 1:**
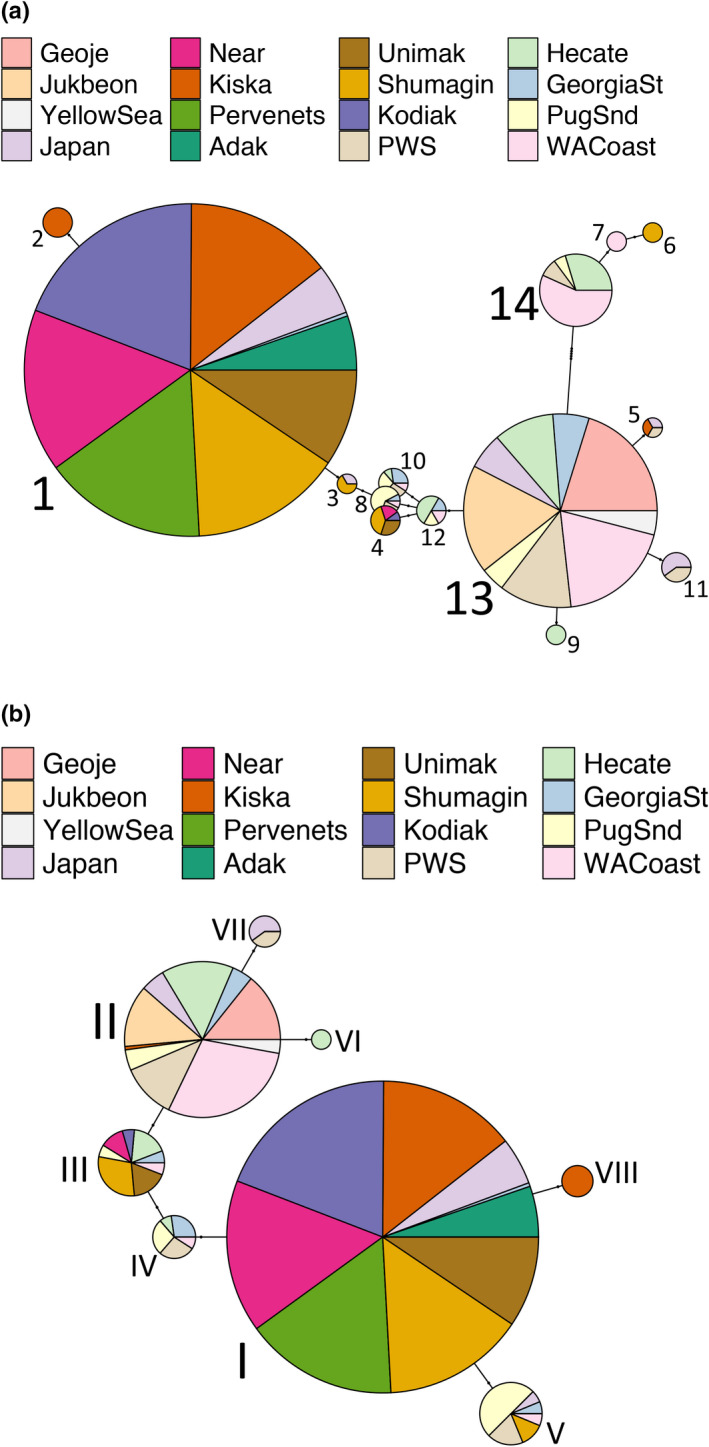
Haplotype network of phased ZP3 sequences of Pacific cod (panel a) and amino acid compositions (panel b). Circles represent haplotypes and pie slices represent contribution by sample collection. The size of each circle is relative to the frequency, although the scale ratio was adjusted for clarity, and the true proportions are shown in Tables 3 and 4. Haplotype and amino acid designations are listed adjacent to each circle and are consistent with designations in Tables 3 and 4, respectively. The Atlantic cod haplotype is identical to haplotype 13 (panel a) and polypeptide II (panel b)

There were 14 unique DNA haplotypes present among all sample collections (Table 3, Table A3). The most frequent, Haplotype 1, was found in over 85% of individuals collected from northern populations the Bering Sea, Aleutian Islands, Shumagin Islands, and Kodiak Island (Table 3, Table A3, Figure 1). Haplotype 1 was also found in 54% of the samples from Japan and a single individual from the Strait of Georgia, but in no other collections. Haplotype 13, which was identical to haplotypes found in Atlantic cod, was the second most commonly observed haplotype, was found in 99 haploid sequences from more southern samples from Korea, Japan, Prince William Sound, Hecate Strait, Washington Coast, Strait of Georgia, and Puget Sound (Figure 1). Haplotype 1 was divergent from Atlantic cod haplotypes and was only found in northern populations. Spatial transitions between these two dominant haplotypes were extremely abrupt, especially in the northeastern Pacific between Kodiak Island and Prince William Sound over a distance of only a few hundred kilometers. Sampling on the west side of the range was more sparse, but the Japan population appeared to be in a mixing zone for ZP3, with multiple haplotypes maintained at intermediate frequencies.

**FIGURE 2:**
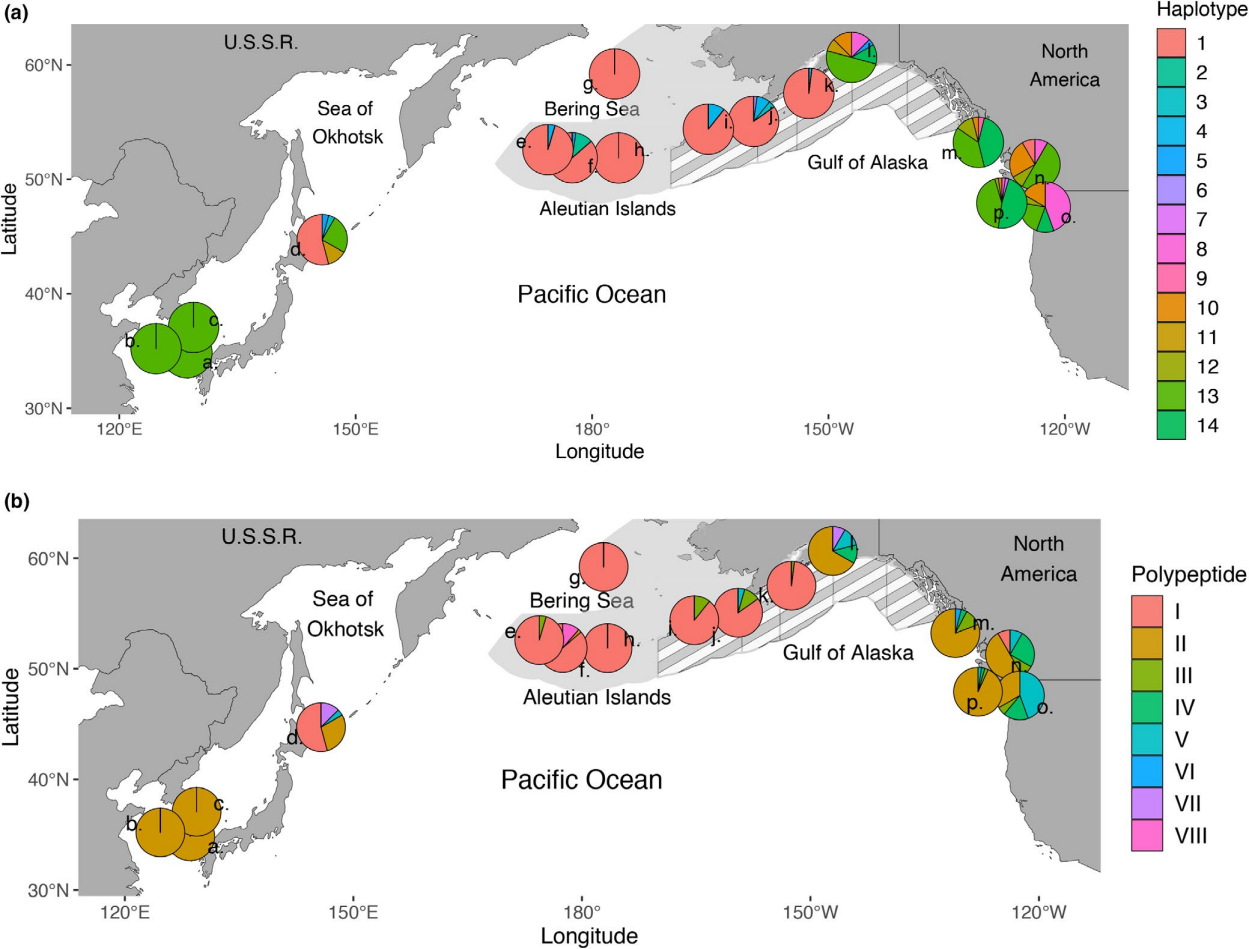
Map with pie charts showing (a) relative haplotype frequencies between all collections, and (b) amino acid sequences based on nonsynonymous changes in the ZP3 DNA‐coding region. The haplotype numbers correspond to Table 3, and roman numerals indicate polypeptide designation, as shown in Table 4. Numbers on map represent the name of the collection, west to east: (a) Geoje, Korea, (b) Yellow Sea, Korea, (c) Jukbyeon, Korea, (d) Sea of Okhotsk, Japan, (e) Near Islands, (f) Kiska Island, (g) Pervenets Canyon, (h) Adak Island, (i) Unimak Island, (j) Shumagin Islands, (k) Kodiak Island, (l) Prince William Sound, (m) Hecate Strait, (n) Strait of Georgia, (o) Puget Sound, (p) Washington Coast. The spatial area comprising the Gulf of Alaska Fishery Management Plan is shown with gray stripes and the area comprising the Bering Sea and Aleutian Islands Fishery Management Plan is light gray

Translation of the coding regions into amino acid sequences resulted in eight segregating polypeptide sequences (Table 4), with the most common (Variant I) present exclusively in Bering Sea, Kodiak Island, Japan, Aleutian Islands, and a single individual from the Strait of Georgia (Table A4, Figure 1b). The second most common polypeptide (Variant II) was the only variant in samples from Korea, and was also observed in samples from the Strait of Georgia, Hecate Strait, Japan, Puget Sound, Prince William Sound, and Washington Coast, as well as a single individual from Kiska (Figure 2b, Table A4). Genotypes for each individual are listed in Table A5.

**TABLE 4:** Segregating sites located in ZP3 exons of Pacific cod (*Gadus macrocephalus*) resulting in nonsynonymous amino acid changes at 1. position 313, 2. position 339, and 3. position 542 bp

Designation	1 (313bp)	2 (339 bp)	3 (542 bp)	*N*
I	Met	Asn	Phe	265
II	Arg	Asp	Tyr	140
III	Arg	Asp	Phe	17
IV	Met	Asp	Phe	11
V	Arg	Asn	Phe	16
VI	Met	Asp	Tyr	1
VII	Arg	Asn	Tyr	5
VIII	Met	Asn	Tyr	5

Note

Designations for each polypeptide are listed and are consistent throughout.

Tests for Hardy–Weinberg equilibrium indicated that genotype frequencies in most of the Eastern Pacific collections deviated significantly from HWE (Prince William Sound, Hecate, Strait of Georgia, and Puget Sound). In contrast, Kiska was the only collection in the Bering Sea that deviated from HWE, and in this collection, 11% of the haplotypes represented a unique variant (Table 5). HWE was not calculable for samples from Korea, as they represented a single haplotype. However, the sample from the Sea of Okhotsk, Japan, was deviated significantly HWE (Table 5).

**TABLE 5:** The number of individuals (*N*), number of distinct haplotypes present (*N*_H_), haplotype diversity (*h*), nucleotide diversity (*π*), observed (*H*_o_) and expected (*H*_e_) heterozygosity, *F*_IS_, and the probability that collections conform to Hardy–Weinberg equilibrium (*p*_HWE_) for each collection. Horizontal lines demarcate collections within regions, and the Bering and E. Pacific collections are summarized (shaded in gray). Ewens–Watterson and Tajima’s *D* results are shown: observed *F* (Obs. *F*), expected *F* (Exp. *F*), Ewens–Watterson probability (E–W *p*), and Slatkin’s test probability (*p*), the number of segregating sites in the sample compared to gadMor3.0 (S), Ramos‐Onsins–Rozas test for detecting population growth (*R*_2_) test with * indicating *p* < .05 and ** indicating *p* > .95, Tajima’s *D*, and the associated *p*‐value under a beta distribution

Location	*N*_H_	*h*	*π*	*H*_e_	*H*_o_	*F*_IS_	*p*_HWE_	Obs. *F*	Exp. *F*	E–W *p*	Slatkin's *p*	*R*_2_	*S*	Tajima's *D* (*p*)
Geoje	1	0	0	0	0	–	–	–	–	–	–	NA	0	–
Yellow Sea	1	0	0	0	0	–	–	–	–	–	–	NA	0	–
Jukbeon	1	0	0	0	0	–	–	–	–	–	–	NA	0	–
Sea of Okhotsk	5	0.6522	0.0036	0.625	0.167	0.733	0.000	0.375	0.376	0.609	0.631	0.175**	4	2.232 (0.027)
Near	2	0.0888	0.0003	0.087	0.091	−0.046	1.000	0.913	0.768	0.791	0.791	0.044*	2	−1.130 (0.268)
Kiska	3	0.2463	0.0006	0.137	0.045	0.672	0.002	0.759	0.610	0.794	0.814	0.107	3	−1.105 (0.280)
Pervenets	1	0	0	0	0	–	–	–	–	–	–	NA	0	–
Adak	1	0	0	0	0	–	–	–	–	–	–	NA	0	–
Unimak	2	0.1984	0.0007	0.191	0.214	−0.120	1.000	0.809	0.755	0.578	0.578	0.099	2	−0.477 (0.675)
Shumagin	4	0.2773	0.0012	0.271	0.304	−0.122	1.000	0.729	0.519	0.886	0.855	0.109	7	−1.543 (0.108)
Kodiak	2	0.0385	0.0001	0.038	0.038	0.000	1.000	0.962	0.785	1.000	1.000	0.137	2	−1.460 (0.133)
(Bering Combined)	6	0.0004	0.1345	0.134	0.104	0.220	0.000	0.866	0.489	0.974	0.969	0.041	8	−1.692 (0.061)
Prince William Snd.	6	0.7246	0.0040	0.694	0.250	0.640	0.040	0.306	0.311	0.579	0.326	0.119	9	−0.301 (0.803)
Hecate	5	0.6831	0.0055	0.657	0.846	−0.288	0.001	0.343	0.387	0.419	0.566	0.211**	7	1.873 (0.070)
St. of Georgia	5	0.7273	0.0028	0.667	0.333	0.501	0.050	0.333	0.304	0.810	0.810	0.180	4	0.466 (0.656)
Puget Sound	5	0.7516	0.0043	0.710	0.111	0.844	0.002	0.290	0.345	0.351	0.292	0.145	8	0.011 (0.966)
WA Coast	6	0.5973	0.0052	0.584	0.727	−0.245	0.627	0.416	0.362	0.741	0.953	0.151	9	1.047 (0.313)
(E Pacific Combined)	10	0.7260	0.0055	0.726	0.525	0.277	0.000	0.280	0.286	0.578	0.781	0.973**	9	−1.908 (0.072)

Note

Dash (–) indicates not applicable.

### 3.3 Sequence variation

**FIGURE 3:**
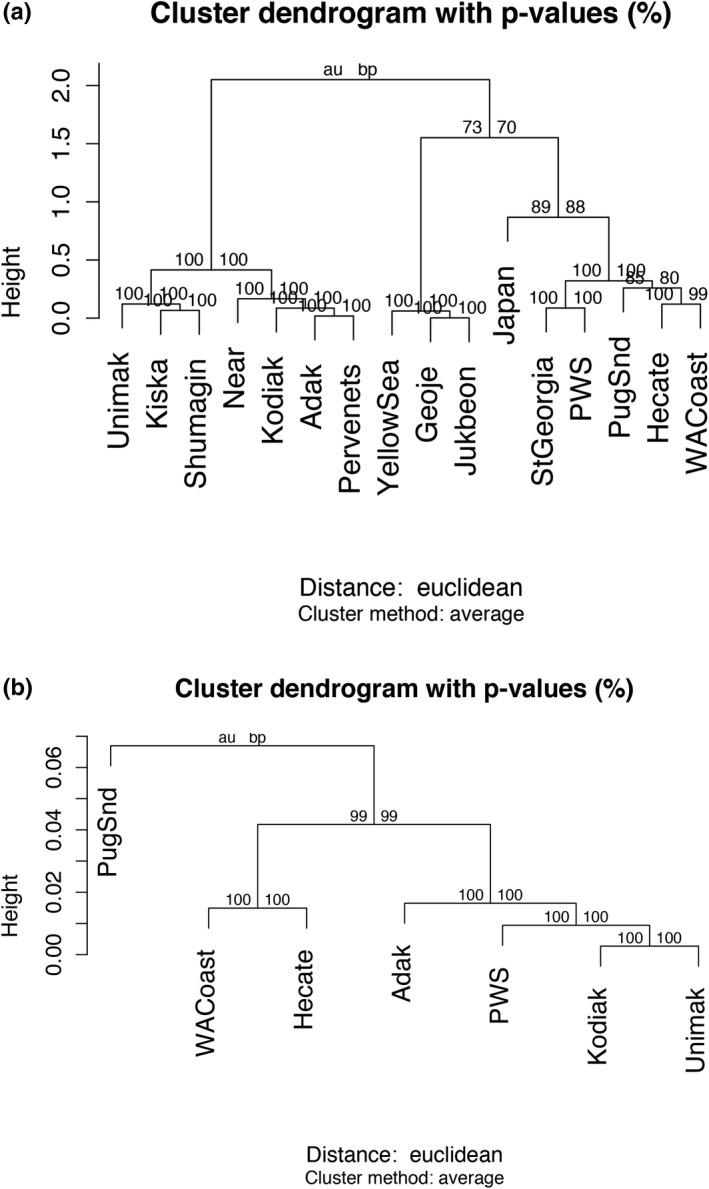
Dendrogram of hierarchical clusters of Pacific cod (*Gadus macrocephalus*) based on pairwise *G*_ST_ for phased ZP3 haplotypes (panel a) and pairwise *F*_ST_ based on previously published RADseq dataset (panel b). Numbers represent probabilities for cluster support based on 10,000 bootstrap replicates, and are presented as AU (left, approximately unbiased) *p*‐values and BP (right, bootstrap probability) values. Sample collections between panels (a) and (b) were the same except for Puget Sound and Unimak, which were sampled in different years, Drinan et al. (2018)

*G*_ST_ was not found to be dependent on sample size (*R* ^2^ = 0.07). Global *G*_ST_ was 0.551, with 95% confidence intervals 0.514–0.588. The dendrogram based on *G*_ST_ at ZP3 (Figure 3a, Table A6) reflected obvious patterns from Figure 2, with more southern populations manifesting high frequencies of haplotype 13 clustering together and more northern populations dominated by haplotype 1 clustering together. Japan, which is the only population that contained high frequencies of both haplotypes 1 and 13, grouped more closely with southern populations. In contrast, neutral RAD loci did not show that break between northern and southern populations, but primarily separated Puget Sound from the rest (Figure 3). The ZP3 break point on the east side of the study was found between Kodiak and Prince William Sound (*G*_ST_ = 0.458, Table A7), whereas RAD data suggested that Prince William Sound and Kodiak are relatively genetically similar at neutral markers (*F*_ST_ = 0.003; Figure 3).

There were no changes in the sequenced region between gadMor3.0 and gadMor_Celtic except a synonymous third position substitution (T in gadMor3.0 and C in gadMor_Celtic) at position 523, which was not variable in Pacific cod. Within Pacific cod sequences, there were five transitions and four transversions (Table 3). Three nonsynonymous substitutions were present in Haplotype 1. Two nonsynonymous substitutions were present in Haplotypes 2, 3, 8, and 10, and one in Haplotypes 4, 9, 11, and 12, which were found in Bering Sea and Eastern Pacific collections (Table A5).

The model with the lowest BIC score was a four‐parameter model with a proportion of invariable sites (I) and a moderate number of parameters, 31 (F81, Felsenstein, 1981). Under this model, Haplotype 13 had the lowest *d*_F81_, indicating the greatest similarity to gadMor3.0, followed by Haplotypes 12 and 5, and then Haplotypes 9 and 11 (Table 3). Haplotype 6 was the most different from Atlantic cod, followed by 7, then 14, 1, 3, and 2.

### 3.4 Statistical tests of selective neutrality

Patterns of selective neutrality varied by region and were not consistent among all tests (Table 5). Large and significant values of Ewens–Watterson homozygosity test (E–W *p*) and the exact test based on Ewens’ sampling theory (Slatkin’s *p*) (>0.9), indicative of directional selection, were observed within the Kodiak collections, and within the combined Bering Sea (Table 5). Slatkin’s *p* was also significant and >0.9 in the WA coast sample (Table 5). All collections from the Bering Sea had negative Tajima’s *D* values, which are consistent with population size expansion, a bottleneck or a selective sweep and/or purifying selection. Conversely, Tajima’s *D* for the Sea of Okhotsk sample was significantly positive, indicative of balancing selection or a reduction in population size. The Ramos‐Onsins–Rozas test for detecting population growth, suggested population declines in the more southerly sites, which were significant in in the Sea of Okhotsk sample, Hecate Strait, and for all Eastern Pacific samples combined (Table 5), and all Western Pacific samples combined (0.243, *p* = .997, not shown in Table 5). The *R*_2_ test showed only one significant increase in population size in the Near Islands sample (Table 5).

### 3.5 Analyses with previously published RADseq dataset

**FIGURE 4:**
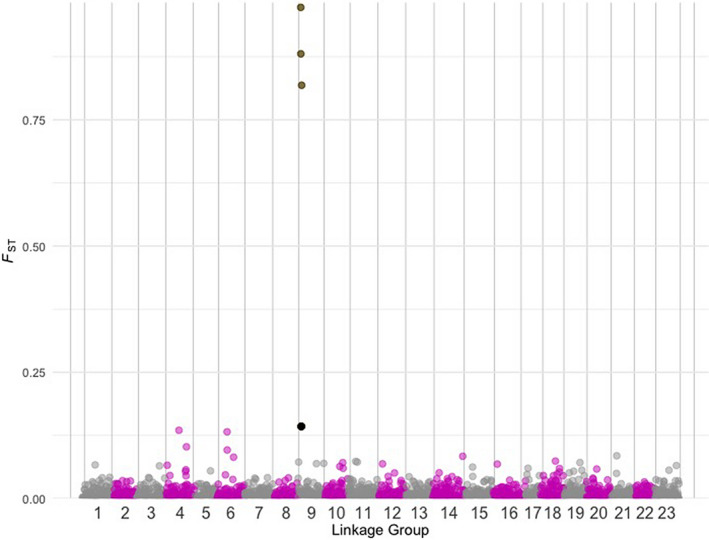
Pairwise *F*_ST_ between Kodiak Island and Prince William Sound samples from the RADseq dataset combined with the remaining eight SNPs (Table 3, all nine SNPs except the SNP in position 2) associated with ZP3 on linkage group 9. Pairwise *F*_ST_ values in black represent the eight SNPs from the current study, and alternating pink and light gray SNPs demarcate linkage group alignment

**FIGURE 5:**
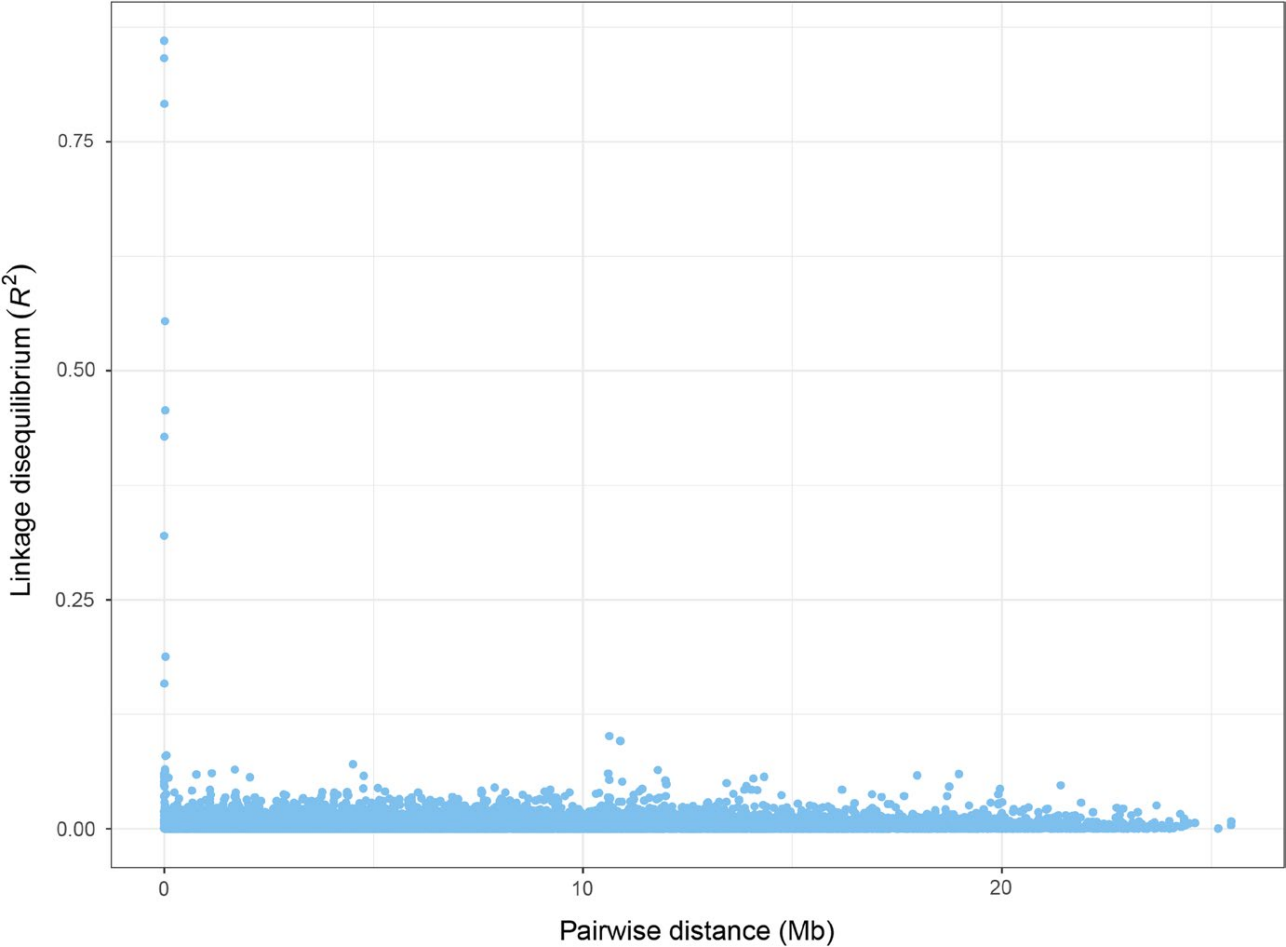
Decay of linkage disequilibrium as a function of the distance separating two SNPs on LG09

In total, we retained 177 SNPs from the RAD data on LG09 after aligning to gadMor3.0 as described. The only SNP with *F*_ST_ > 0.01 was found in the short read sequence identified in Drinan et al. that aligned to the predicted ZP3 gene (2018; Table A1). Pairwise *F*_ST_ between Hecate Strait and Kodiak Island samples show that the SNP associated with ZP3 is a high‐*F*_ST_ outlier, *F*_ST_ = 0.3 (Figure 4). In this comparison, it has the highest *F*_ST_ across the genome, followed by another high *F*_ST_ SNP on LG06 (*F*_ST_ = 0.2). The global *F*_ST_ associated with that SNP was 0.71. Linkage disequilibrium decayed quickly along LG09, and only seven SNPs that were <0.025 Mb apart had *R* ^2^ values > 0.20 (Figure 5), indicating that there were no major inversions within this linkage group.

## 4 DISCUSSION

Multiple studies have documented neutral genetic diversity and limited gene flow in Pacific cod, but the extent of local adaptation is currently unknown. Our goal was to assess spatial variation in ZP3, a gene that is putatively under strong selection (Drinan et al., 2018), to understand whether there was evidence for selection across a large geographic range and to investigate potential mechanisms for this selection. Significant recent declines have occurred in this species due to anomalously warm conditions in the Gulf of Alaska, and results were intended to assist in conservation and management of these stocks. We hypothesized that patterns of population structure at the ZP3 gene may differ from genetic markers in the rest of the genome, and show evidence for directional selection. We also anticipated that patterns of diversity in ZP3 might provide insight into its functional significance.

Our first significant finding was striking spatial differences based on DNA sequence data of the ZP3 variable region that suggest limited gene flow at this locus. DNA sequence variation clustered into two broad geographic groups with almost no overlap in haplotypes: northern samples within and adjacent to the Bering Sea, and collections further south (Figure 2). Coding sequences found in the Bering Sea group (collections from the Aleutian Islands, Bering Sea, Shumagin Islands, and Kodiak Island collections) in the north were replaced by an almost completely different and diverse set of coding sequences from Prince William Sound and southward. The inclusion of the Kodiak Island and Shumagin Islands collections in the Bering Sea group is notable from a management perspective, as the US federal fishery for Pacific cod currently includes Kodiak Island and the Shumagin Islands region in the Gulf of Alaska management area (Figure 3; NPFMC, 2020). Management quotas are set using abundance indices from summer National Marine Fisheries Service surveys, while samples in this study were based on winter spawning distributions. Seasonal changes in the distribution of northern and southern stock components would, therefore, not only affect overall quotas but may also differentially affect summer and winter fisheries in the Gulf of Alaska. High‐throughput assays for large‐scale screening of samples from fisheries and surveys are therefore needed for a spatiotemporal analysis of stock boundaries within the Gulf of Alaska. On the western side of the Pacific Ocean, there appeared to be a transition region north of Japan where northern and southern haplotype co‐occurred (Figure 3). Samples from Korea exhibited only a single coding sequence type, and could be considered a third subgroup (Polypeptide II, Figure 2).

Our second main finding was high genetic differentiation in ZP3 compared to the rest of the genome (Figure 4) and multiple lines of evidence for divergent selection in ZP3 in the Bering Sea collection. The highest number of nonsynonymous substitutions was apparent in the prevalent Bering Sea haplotype 1, with d*_N_*/d*_S_* = 3, and large d*_N_*/d*_S_* ratios of this magnitude have been associated with divergent lineages (Kryazhimskiy & Plotkin, 2008). Local adaptation is likely responsible for the geographic pattern of coding sequence haplotypes coupled with high levels of differentiation. The lack of linkage disequilibrium (Figure 5), negative Tajima’s *D* values, and significant departures from HWE (Table 5) associated with ZP3 SNPs support the conclusion that the ZP3 gene is under selection. The lack of linkage disequilibrium also indicates selection rather than hitchhiking associated with a nearby gene (Figure 5).

In samples with high diversity, *F*_IS_ was remarkably high, and there was a notable lack of heterozygotes in several collections, such as Prince William Sound, Strait of Georgia, Kiska, and Puget Sound (Table 5). In addition, there were no heterozygotes for the two most common haplotypes, Haplotypes 1 and 13 (Table A5). This deviation from Hardy–Weinberg equilibrium may suggest that the two haplotype groups mate assortatively, with very little gene flow between them. However, additional experimental studies are required to test this notion.

While one premise of this paper was to examine the putative ZP3 gene for evidence of selective diversification, the gene function, especially in relation to gamete recognition and temperature tolerance, cannot be tested without laboratory experimentation. Such studies would help provide insight into the implications of diversification of ZP3 in Pacific cod. A greater understanding of the role of thermal adaptation for the productivity and persistence of Pacific cod would be beneficial to management. ZP3 appears to have undergone neofunctionalization in fish species as antifreeze glycoproteins for cold‐water adaptation (Cao et al., 2016; Conner & Hughes, 2003). Experiments have shown that zona pellucida proteins can noncolligatively lower the freezing and melting point of a solution (Cao et al., 2016). Zona pellucida paralogues as antifreeze glycoproteins are expressed in the exocrine pancreas in adult fishes, circulated throughout the body, and may aid in reducing body ice load (Cao et al., 2016). The collections in the Bering Sea group were situated within or adjacent to the southeastern Bering Sea, an ecosystem structured by cold pool water <2°C that remains following the retreat of the Bering Sea ice sheet in spring (Stevenson & Lauth, 2019). Residence within colder water may be consistent with selective adaptation to colder temperatures via ZP3 as antifreeze glycoproteins, but further testing is needed to substantiate this hypothesis. The narrow thermal window (3–6°C) of successful hatching in Kodiak Island cod may be in part responsible for the high mortality in the Gulf of Alaska during recent marine heat waves (Laurel & Rogers, 2020). Given the amino acid differences between different ZP3 haplotypes, further investigation of the significance of this genetic variation for tolerated thermal windows may be of interest for management and conservation under climate change. Demographic trajectories of cod in different parts of the Gulf of Alaska support the difference in temperature tolerance within the Gulf of Alaska. During and after the heat wave in the Gulf of Alaska (2014–2016), the population size of the Eastern Gulf of Alaska cod stock remained low but stable (Barbeaux, Holsman, et al., 2020). In contrast, the cod stock in the Central Gulf of Alaska declined by about 30% per year, and by 23% per year in the Western Gulf of Alaska. Although these population trajectories are consistent with different thermal tolerances related to ZP3 haplotypes, this hypothesis remains to be tested.

In Atlantic cod, genetic divergence among ecotypes has been shown occur within large genomic inversions on linkage groups 1, 2, 7, and 12, each of which span several Mb (Berg et al., 2017; Kirubakaran et al., 2016). While RADseq data are not powerful for identifying small genomic inversions, larger inversions would cause additional *F*_ST_ outliers and linkage disequilibrium extending over greater genomic distances (Huang et al., 2020; Petrou et al., 2021). Here, only one SNP out of 177 SNPs on linkage group 9 had an *F*_ST_ over 0.01, and linkage disequilibrium was minimal between loci separated by more than a few thousand base pairs, suggesting that inversions likely do not play a role in the striking genetic differentiation observed at the ZP3 gene. ZP3 was identified as the largest *F*_ST_ outlier, and further work is needed to resolve whether it resides within a genomic island of divergence or a small inversion. Nevertheless, our results provide insight into a distinction between adaptive differentiation in Pacific cod and Atlantic cod. The specific mechanism driving selection and diversification in Pacific cod certainly merits further research.

Our data are consistent with a demographic movement scenario as follows: Pacific cod likely originated in the Pacific Ocean from an ancestor to Atlantic cod that invaded the Pacific ~4 Million years ago (Árnason & Halldórsdóttir, 2019). Oceanographic events of the Pleistocene likely forced extant Pacific cod southward. Pacific cod with ZP3 haplotype 13 identical to the warm‐ and cold‐adapted Atlantic cod counterparts are currently found in the southern part of the range (Table 3). During Pleistocene glaciations, water levels fluctuated and the Bering Sea shelf was exposed during several time periods, forming the Bering land bridge as recently as 30,000–18,000 years ago (Elias et al., 1996; Hopkins, 1967). Glaciers extended from the Alaska Peninsula to the Aleutian chain through the southern extent of Alaska to northwestern Canada southward to Puget Sound (Batchelor et al., 2019). As cod recolonized the north Pacific, Aleutian Islands, and Bering Sea (Canino et al., 2010), mutations leading to the common haplotype 1 likely occurred in the putative ZP3 gene. The Bering Sea in the northernmost extent of the Pacific Ocean contains a deep basin <3500 m to the southwest and continental shelf <200 m to the northeast (Stabeno et al., 1999), and is currently habitat for the largest spawning population of Pacific cod within its range (Spies et al., 2020).

The extreme differentiation among Pacific cod populations at ZP3 with almost no shared haplotypes between groups suggests an almost complete isolation of cod in eastern and western Gulf of Alaska. This finding is hard to reconcile with the lack of differentiation elsewhere in the genome. An alternative explanation for our finding would be that the different haplotypes of ZP3 actually represent paralogous loci that amplified differentially between the two populations. Indeed, Antarctic fishes may have up to 35 copies of ZP genes that occur as single copies in temperate fishes (Cao et al., 2016). It is, therefore, not impossible that the observed differentiation represents copy number variation among Pacific cod populations, similar to that observed in American lobster *Homarus americanus* (Dorant et al., 2020) and capelin *Mallotus villosus* (Cayuela et al., 2020). However, this explanation seems unlikely, because (i) a series of different primers were tested, with identical results, and (ii) there was no evidence for sequencing ambiguities other than those in heterozygotes. In any case, even if the variation we observed was copy number variation rather than allelic variation, it would demonstrate a genetic difference between these two groups that is readily identifiable and likely functionally significant. Nevertheless, for accurate functional analysis, the possibility of gene duplication should be further investigated.

The ability of Pacific cod to adapt in a changing climate may depend on the depth of genetic variation acquired through its complex evolutionary history (Árnason & Halldórsdóttir, 2019). This work provided evidence for the maintenance of different coding sequence haplotypes of the putative ZP3 gene through selection in Pacific cod. Results were consistent with directional selection in the Bering Sea (Bering Sea, Aleutian Islands, Shumagin Islands, and Kodiak Island), and large regional differences among ZP3 haplotype frequencies between the Bering Sea group and regions further south. Results were also indicative of selection currently acting on northern collections, and may indicate local adaptation driven by differences in ZP3. Our work supports the idea that Korea and the eastern North Pacific were once glacial refugia for Pacific cod, based on the low diversity in Korean samples and the presence of variants that appear most similar to Atlantic cod. While further work is needed to understand the functional advantage of selected types of ZP3 in Pacific cod, we hypothesize that its function in thermoregulation and sperm‐binding protein in other species may provide clues to its significance in Pacific cod.

## CONFLICT OF INTEREST

The authors declare that they have no conflicts of interest.

## AUTHOR CONTRIBUTIONS

**Ingrid Spies:** Conceptualization (equal); Data curation (lead); Formal analysis (lead); Funding acquisition (lead); Investigation (lead); Methodology (lead); Project administration (lead); Writing‐original draft (lead). **Daniel P Drinan:** Conceptualization (equal); Methodology (equal). **Eleni Leto Petrou:** Investigation (equal); Methodology (equal); Visualization (equal); Writing‐review & editing (equal). **Rory Spurr:** Data curation (equal); Investigation (equal); Methodology (equal). **Carolyn Tarpey:** Data curation (equal); Formal analysis (equal); Writing‐review & editing (equal). **Theodore Hartinger:** Conceptualization (equal); Data curation (equal); Formal analysis (equal). **Wesley A. Larson:** Formal analysis (equal); Writing‐review & editing (equal). **Lorenz Hauser:** Conceptualization (equal); Project administration (equal); Writing‐review & editing (equal).

## Data Availability

The data that support the findings of this study are openly available in GenBank at https://www.ncbi.nlm.nih.gov/genbank/, accession numbers MW468336–MW468402. In addition, the phased DNA sequence file is available at Dryad https://doi.org/10.5061/dryad.wdbrv15q7.

## APPENDIX 1

**TABLE A1:** Short read sequence (95bp) associated with the highest *F_ST_* based on RAD sequencing (Drinan et al., 2018)

5' TGCAGGAGGCATAACGTGAGCAGTCTGGCCCTGGACCCGATCTTCA CCCCGTTCTCGGCCATCAAAGTGTCCGAGGAGCTTTTGCACTTCAGTTT 3'

**TABLE A2:** Primer sets ZP_L1, ZP_L2, ZP_L3, and ZP_L4 were used to screen for variation across the DNA sequence coding for the zona pellucida sperm‐binding protein 3 in samples of Pacific cod (*Gadus macrocephalus*)

Primer name	Forward	Reverse	*T*_M_ (°C)	*F* pos.	*R* pos.	Exon
ZP_L1	gagcccgctagatcacttgt	tgtaatctggtgggcagcta	56.5	1	592	1
ZP_L2	agctgcccaccagattacat	gattgcgtgcgctatactga	62.5	574	1372	2,3,4
ZP_L3	aatgtttgtttccgcactca	ccgcatacaaacacacacat	57	1416	1766	5,6
ZP_L4	ttgcaggcttcaaaaagtca	catcatagcaagggcatgaa	56	3223	3242	8
ZP_GM	gcaatctgagggtaggacca	aacgcagtgatccacaaaga	58	612	1153	2,3,4

Note

Primer set GP_GM was used to amplify a variable region. The annealing temperature, *T*_M_ (°C), used in polymerase chain reaction is shown for each primer set. The position of the forward and reverse primer on the gadMor3.0 genome sequence is shown, as well as which exons are included in the intervening sequence.

**TABLE A3:** Frequency of observed nucleotide haplotides in sample collections of Pacific cod (*Gadus macrocephalus*)

No.	Geoje	Jukbeon	YS	Japan	Near	Kiska	Perv.	Adak	Unimak	Shum.	Kodiak	PWS	Hecate	Georgia	PS	WA	Tot.
1	0	0	0	13	42	38	42	14	25	39	51	0	0	1	0	0	265
2	0	0	0	0	0	5	0	0	0	0	0	0	0	0	0	0	5
3	0	0	0	1	0	0	0	0	0	2	0	0	0	0	0	0	3
4	0	0	0	0	2	0	0	0	3	4	1	0	0	0	0	0	10
5	0	0	0	1	0	1	0	0	0	0	0	1	0	0	0	0	3
6	0	0	0	0	0	0	0	0	0	1	0	0	0	0	0	0	1
7	0	0	0	0	0	0	0	0	0	0	0	0	0	0	0	1	1
8	0	0	0	0	0	0	0	0	0	0	0	3	0	1	8	1	13
9	0	0	0	0	0	0	0	0	0	0	0	0	1	0	0	0	1
10	0	0	0	0	0	0	0	0	0	0	0	3	1	3	3	1	11
11	0	0	0	3	0	0	0	0	0	0	0	2	0	0	0	0	5
12	0	0	0	0	0	0	0	0	0	0	0	0	3	1	1	1	6
13	20	18	4	6	0	0	0	0	0	0	0	12	10	6	4	19	99
14	0	0	0	0	0	0	0	0	0	0	0	3	11	0	2	21	37

Note

The Haplotype number (No.) from 1 to 14 is shown with the number of inferred haplotypes from each sample collection (Georgia = Strait of Georgia, Hecate = Hecate Strait, Perv. = Pervenets, PS = Puget Sound, Shum. = Shumagin Islands, WA = Washington Coast, YS = Yellow Sea, Japan = Sea of Okhotsk).

**TABLE A4:** Frequency of segregating polypeptide sequences in sample collections of Pacific cod (*Gadus macrocephalus*)

NNo.	Geoje	YS	Jukbeon	Japan	Near	Kiska	Perv.	Adak	Unimak	Shum.	Kodiak	PWS	Hecate	Georgia	PS	WA	Tot.
I	0	0	0	13	42	38	42	14	25	39	51	0	0	1	0	0	265
II	20	4	18	7	0	1	0	0	0	0	0	16	21	6	6	41	140
III	0	0	0	0	2	0	0	0	3	5	1	0	3	1	1	1	17
IV	0	0	0	0	0	0	0	0	0	0	0	3	1	3	3	1	11
V	0	0	0	1	0	0	0	0	0	2	0	3	0	1	8	1	16
VI	0	0	0	0	0	0	0	0	0	0	0	0	1	0	0	0	1
VII	0	0	0	3	0	0	0	0	0	0	0	2	0	0	0	0	5
VIII	0	0	0	0	0	5	0	0	0	0	0	0	0	0	0	0	5

Note

The Variant number (No.) from I through VIII is shown with the number of inferred polypeptide sequences from each sample collection (Georgia = Strait of Georgia, Hecate = Hecate Strait, Perv. = Pervenets, PS = Puget Sound, Shum. = Shumagin Islands, WA = Washington Coast, YS = Yellow Sea, Japan = Sea of Okhotsk).

**TABLE A5:** Diploid genotypes by collection of Pacific cod (*Gadus macrocephalus*), consisting of Haplotype A/Haplotype B, followed by the number of individuals (in parentheses) in which it was observed

Collection	Haploid genotypes (frequency)
Geoje	13/13 (10)
Yellow Sea	13/13 (2)
Jukbeon	13/13 (9)
Sea of Okhotsk	1/1 (6), 1/5 (1), 3/11 (1), 11/11 (1), 13/13 (3)
Near Islands	1/1 (20), 1/4 (2)
Kiska Island	1/1 (19), 2/2 (2), 2/5 (1)
Pervenets Canyon	1/1 (21)
Hecate Strait	8/12 (1), 9/13 (1) 12/13 (2), 13/14 (7), 14/14 (2)
Adak Island	1/1 (7)
Unimak Pass	1/1 (11), 1/4 (3)
Shumagin Islands	1/1 (16), 1/3 (2), 1/4 (4), 1/6 (1)
Kodiak Island	1/1 (25), 1/4 (1)
Prince William S.	5/8 (1), 8/8 (1), 10/10 (1), 11/11 (1), 10/13 (1), 13/13 (5), 13/14 (1), 14/14 (1)
Puget Sound	8/8 (1), 10/10 (4), 8/12 (1), 13/13 (2), 14/14 (1)
Washington Coast	7/14 (1), 8/14 (1), 10/14 (1), 12/13 (1), 13/13 (3), 13/14 (12), 14/14 (3)

**TABLE A6:** Pairwise *G*_ST_ among collections of Pacific cod (*Gadus macrocephalus*): Georgia = Strait of Georgia, PWS = Prince William Sound, PugSnd = Puget Sound, WACoast = Washington Coast

	Adak	Geoje	Georgia	Hecate	Japan	Jukbeon	Kiska	Kodiak	Near	PWS	Pervenets	PugSnd	Shumagin	Unimak	WACoast
Geoje	1.0000														
Georgia	0.4511	0.1837													
Hecate	0.4951	0.2939	0.0720												
Japan	0.1753	0.4021	0.1090	0.1604											
Jukbeon	1.0000	0.0000	0.1830	0.2932	0.4015										
Kiska	0.0505	0.7816	0.3314	0.3739	0.0940	0.7813									
Kodiak	−0.0018	0.9623	0.4351	0.4789	0.1643	0.9623	0.0418								
Near	0.0113	0.9154	0.4080	0.4518	0.1435	0.9152	0.0293	0.0002							
PWS	0.4735	0.1691	0.0041	0.0421	0.1231	0.1684	0.3553	0.4580	0.4316						
Pervenets	0.0000	1.0000	0.4560	0.4997	0.1812	1.0000	0.0565	0.0043	0.0174	0.4782					
PugSnd	0.4635	0.3617	0.0609	0.0997	0.1599	0.3611	0.3470	0.4483	0.4222	0.0520	0.4683				
Shumagin	0.0457	0.7573	0.3173	0.3595	0.0841	0.7570	0.0169	0.0332	0.0156	0.3417	0.0518	0.3331			
Unimak	0.0431	0.8219	0.3535	0.3966	0.1074	0.8217	0.0216	0.0257	0.0061	0.3778	0.0491	0.3688	−0.0021		
WACoast	0.5398	0.3131	0.0935	−0.0011	0.1844	0.3124	0.4115	0.5221	0.4936	0.0506	0.5441	0.1178	0.3964	0.4355	
YellowSea	1.0000	0.0000	0.1579	0.2697	0.3798	0.0000	0.7712	0.9604	0.9110	0.1435	1.0000	0.3386	0.7459	0.8132	0.2893

**TABLE A7:** Models of nuclear evolution and the associated AIC, AICc, and BIC values

Model	df	logLik	AIC	AICc	BIC
F81+I	31	−965.80	1993.60	1997.49	2126.81
JC+I	28	−976.57	2009.15	2012.31	2129.47
F81+G+I	32	−964.24	1992.48	1996.62	2129.98
JC+G+I	29	−975.01	2008.02	2011.42	2132.64
HKY+I	32	−965.67	1995.35	1999.49	2132.85
F81+G	31	−970.13	2002.25	2006.14	2135.46
K80+I	29	−976.48	2010.97	2014.36	2135.58
HKY+G+I	33	−964.10	1994.20	1998.61	2136.00
JC+G	28	−980.88	2017.76	2020.92	2138.08
K80+G+I	30	−974.92	2009.84	2013.47	2138.75
HKY+G	32	−970.03	2004.06	2008.20	2141.57
SYM+I	33	−967.42	2000.84	2005.25	2142.64
GTR+I	36	−958.46	1988.92	1994.19	2143.62
SYM+G+I	34	−964.91	1997.83	2002.51	2143.93
K80+G	29	−980.81	2019.62	2023.01	2144.24
GTR+G+I	37	−956.26	1986.51	1992.08	2145.50
F81	30	−979.91	2019.82	2023.45	2148.73
JC	27	−990.64	2035.28	2038.22	2151.31
SYM+G	33	−972.08	2010.17	2014.57	2151.97
GTR+G	36	−963.08	1998.17	2003.43	2152.86
HKY	31	−979.82	2021.64	2025.53	2154.85
K80	28	−990.58	2037.16	2040.32	2157.48
SYM	32	−982.10	2028.20	2032.34	2165.71
GTR	35	−973.00	2016.00	2020.97	2166.40
